# Hepatitis B and Hepatitis C Infection Biomarkers and TP53 Mutations in Hepatocellular Carcinomas from Colombia

**DOI:** 10.1155/2011/582945

**Published:** 2011-10-31

**Authors:** Maria-Cristina Navas, Iris Suarez, Andrea Carreño, Diego Uribe, Wilson Alfredo Rios, Fabian Cortes-Mancera, Ghyslaine Martel, Beatriz Vieco, Diana Lozano, Carlos Jimenez, Doriane Gouas, German Osorio, Sergio Hoyos, Juan Carlos Restrepo, Gonzalo Correa, Sergio Jaramillo, Rocio Lopez, Luis Eduardo Bravo, Maria Patricia Arbelaez, Jean-Yves Scoazec, Behnoush Abedi-Ardekani, Regina M. Santella, Isabelle Chemin, Pierre Hainaut

**Affiliations:** ^1^Grupo de Gastrohepatologia, Facultad de Medicina, Universidad de Antioquia, Medellín, Colombia; ^2^International Agency for Research on Cancer, 150 Cours Albert Thomas, 69372 Lyon, France; ^3^Departamento de Patología, Facultad de Medicina, Universidad de Antioquia, Medellín, Colombia; ^4^Hospital Pablo Tobón Uribe, Medellín, Colombia; ^5^Departamento de Patologia, Fundacion Santa Fe de Bogota, Bogotá D.C., Colombia; ^6^Departamento de Patología, Facultad de Salud, Universidad del Valle, Cali, Colombia; ^7^Grupo de Epidemiologia, Facultad Nacional de Salud Publica, Universidad de Antioquia, Medellín, Colombia; ^8^Service d'Anatomie Pathologique, Hôpital Edouard Herriot, 69437 Lyon, France; ^9^Mailman School of Public Health, Columbia University, NY 10032, USA; ^10^INSERM U871, 69424 Lyon, France

## Abstract

Hepatocellular Carcinoma (HCC) is a leading cause of cancer-related death worldwide. Globally, the most important HCC risk factors are Hepatitis B Virus (HBV) and/or Hepatitis C Virus (HCV), chronic alcoholism, and dietary exposure to aflatoxins. We have described the epidemiological pattern of 202 HCC samples obtained from Colombian patients. Additionally we investigated HBV/HCV infections and *TP53* mutations in 49 of these HCC cases. HBV biomarkers were detected in 58.1% of the cases; HBV genotypes F and D were characterized in three of the samples. The HCV biomarker was detected in 37% of the samples while HBV/HCV coinfection was found in 19.2%. Among TP53 mutations, 10.5% occur at the common aflatoxin mutation hotspot, codon 249. No data regarding chronic alcoholism was available from the cases. In conclusion, in this first study of HCC and biomarkers in a Colombian population, the main HCC risk factor was HBV infection.

## 1. Introduction

Primary liver cancer is the third leading cause of cancer death. Moreover, it is the fifth and eighth most frequent cancer among men and women worldwide, respectively. The most common histological type of liver cancer is hepatocellular carcinoma (HCC) accounting for 80 to 90% of the cases [1].

HCC incidence is highly variable among geographic regions depending on the prevalence of risk factors and the incidence of liver cirrhosis; actually, 70 to 90% of HCC cases develop from cirrhotic liver. Major risk factors of HCC include Hepatitis B Virus (HBV) and/or Hepatitis C Virus (HCV) infection and heavy alcohol consumption. In fact, chronic HBV and HCV infections have been recognized as liver carcinogens with an imputable fraction of at least 75% of HCC cases; moreover, it has been estimated that HBV is responsible for 50 to 80%, whereas HCV is associated to 10 to 25% of HCC cases. Other environmental and genetic HCC risk factors include dietary exposure to aflatoxins, diabetes, obesity, nonalcoholic steatohepatitis, and hereditary hemochromatosis [1–3].

The burden of HCC is growing in different continents. Central and South America were in the past known as low-incidence liver cancer regions. However, according to the last published GLOBOCAN analysis, the incidence rates of liver cancer in these countries correspond to low and intermediate incidence.

Colombia is a country of relatively low incidence of liver cancer with incidences of primary liver and bile duct cancers of 3.1/100,000 in males and 2.7/100,000 in females. However, there is only one active cancer registry in the country, based in Cali city, an urban area; nevertheless, whether this situation is representative for the country as a whole is unknown [4]. Additionally, the national mortality registry reported around 1,300 deaths from malignant liver and intrahepatic bile ducts cancer that corresponds to a mortality rate of 3.23 and 3.09/100 000 in men and women, respectively [5]. So far, there is no study assessing the geographic variations in incidence or risk factor of chronic liver disease and liver cancer in Colombia. 

Latin American data about HCC risk factors are limited. The first recent prospective study of HCC etiology in 9 Latin American countries showed that the primary risk factor was chronic HCV infection (30.8%), followed by chronic alcoholism (20.4%), and chronic HBV infection (10.8%) [6]. Although HCV infection is the most important HCC risk factor in Argentina, Mexico, and Brazil, regional differences have been described between northern and southern states in Brazil. Indeed, HBV infection is the most prevalent risk factor in northern states in Brazil, as in Peru [7–14].

According to the World Health Organization, Colombia has a moderate endemicity for HBV; although there are several epidemiological patterns given the geographic, ethnic, cultural, and socioeconomic status of the population. Data from the Colombian National Institute of Health indicate that, in 2007, a seroprevalence of HBsAg of 0.27% (range 0.08–1.27) was found in 1573 blood bank samples from across the country. In some rural areas, such as Amazonas state, rates of chronic HBV carriage over 5% have been reported [15, 16]. 

Although the prevalence of HCV infection in the general population in Colombia is unknown, the WHO estimates a prevalence between 1 to 2.5% for this country, considering the data from the National Blood Banks Unit of the Colombian National Institute of Health. Indeed, while the seroprevalence of HCV in blood donors was 0.7–1% in 1993–1996 and 0.5% in 2002, in a cohort of 500 multitransfused patients recruited from the two largest cities in Colombia, Bogota and Medellin, the HCV prevalence was 9% [17].

Data on exposure to aflatoxins, a class of mycotoxin contaminating traditional foodstuff in tropical countries, are even scarcer [18]. A survey of aflatoxin contamination in selected Colombian foods was conducted over a 12-month period on a total of 248 samples collected in supermarkets, retail stores, and stock centres [19]. Aflatoxins were detected in 22 samples, including 14 of 109 samples of corn and corn products and 4 of 40 samples of rice and rice products. Twelve of the 22 positive samples exceeded the maximum tolerable level of AFB1 adopted by most countries (5 ng/g), including 10 samples of corn and corn products. Given that corn is part of the common diet of Colombian inhabitants; it is likely that AFB1 may represent a significant exposure at least in a fraction of the population. Finally, the role of chronic alcoholism (mostly in the form of cane sugar alcohol) may also be significant [20–23]. 

In the present study, we describe the sociodemographic variables of 202 HCC cases, who attended four reference institutions in Colombia during the period 2000–2007, and, for the first time, the prevalence of biomarkers in a series of 49 HCC cases. We report that the HBV biomarker was detectable in 58.1% of the cases and the HCV biomarker in 37%. Among TP53 mutations, 10.5% occur at the common aflatoxin mutation hotspot, codon 249, although G12457T (exon 5) and G13804A (exon 8) were present in 2.9% of the HCC samples. Unfortunately, data on chronic alcoholism was not available from the cases. These results suggest that the principle HCC risk factor in this Colombian population is HBV infection and low to moderate of AFB1 exposure.

## 2. Materials and Methods

### 2.1. Liver Samples

HCC samples were obtained from archived cases in the Departments of Pathology of four institutions in the three largest cities in Colombia, Bogota, Medellin, and Cali, during the period from 2000 to 2007. The institutions correspond to Fundacion Santa Fe de Bogota (47 cases), Hospital Pablo Tobon Uribe (31 cases), Facultad de Medicina, Universidad de Antioquia (114 cases), and Hospital Universitario del Valle (10 cases).

From 202 HCC cases registered at the archives, 49 paraffin-fixed liver samples were available for immunochemistry and molecular biology assays. The histological pattern and grade of tumor differentiation (Edmonson and Steiner grading system) was assigned by two independent pathologists.

### 2.2. Immunochemistry Analysis

#### 2.2.1. p53

The isoforms of p53 were detected on deparaffinized tissue sections using standard protocols with CM1 antibody (rabbit polyclonal immunoglobulin G antihuman p53, 1/500, Novacastra Laboratories Ltd., Newcastle, UK). The antibodies were detected using biotinylated immunoglobulin G, streptavidine-peroxidase, and diaminobenzidine-based detection (Vector Laboratories, Inc., Burlingame, Calif, USA).

#### 2.2.2. HBx and Core HCV

After deparaffinization and rehydratation antigen retrieval was applied by vaporizer in Target Retrieval Solution pH 6.0 (Dako). As primary antibody Core HCV monoclonal antibody (anti-Core HCV aa70–90 CHEMICON, Millipore) or HBx monoclonal antibody Hepatitis B Virus X-Protein (Trans-Activator X Gene Product) monoclonal antibody (Clon 227, CHEMICON, International, Inc.) were used at a dilution 1 : 50. The kit ultravision LP detection System HRP Polymer and DAB Plus Chromogen (Lab Vision corporation) was used for the detection of HBV and HCV antigens.

#### 2.2.3. AFB1-DNA Adducts

The standardization of adducts detection was performed using liver tissue sections from rats treated with AFB1. The liver tissue sections were labeled using the antibody highly specific for AFB1-Fapy adducts 6A10, developed and characterized by Hsieh et al. [24]. Briefly, the sections were first treated with 5 mM Na_2_CO_3_/30 mM NaHCO_3_ (pH 9.0) to open the guanine adducts ring. The antigen retrieval was performed using citrate buffer pH 6.0 (Dako). The liver sections were then treated with RNase A (100 *μ*L/mL, Fermentas, *RNase A*,* DNase*,* and Protease-free*), with Proteinase K for 10 min at 37°C (10 *μ*L/mL, Gentra Puregene) and with NaOH 50 mM in 40% ethanol for DNA denaturation. Slides were then incubated with the antibody 6A10 at a dilution 1 : 20 at 4°C overnight. The reaction was detected using the kit ultravision LP detection System HRP Polymer & DAB Plus Chromogen (Lab Vision Corporation). 

#### 2.2.4. DNA Extraction

DNA was extracted from 4 *μ*m unstained paraffin sections. The sections were deparaffined in xylene and ethanol. Then, the tumor tissue areas of interest were scrapped into 1.5 mL sterile microcentrifuge. DNA was extracted using QIAmp DNA Micro kit (Qiagen, Hilden, Germany), according to the manufacturer's instructions. DNA extracts were stored at −20°C. 

#### 2.2.5. TP53 Mutations

DNA was used for amplification of exon 7 of *TP53* gene with the primers flanking the exon (sense-333 ACTTGCCACAGGTCTCCCCAA and antisense-313 AGGGGTCAGCGGCAAGCAGA) as described elsewhere [25]. Briefly, the PCR was carried out in a volume of 25 *μ*L containing 5 *μ*L of DNA, 1 U of Platinum *Taq* DNA polymerase High Fidelity (Invitrogen Carlsbad, USA), 0.4 *μ*M of each primer, dNTP (200 *μ*M each), 1X High Fidelity Buffer Taq polymerase (Invitrogen), 0.5 mM of MgSO_4_ (Invitrogen), and nucleases-free water (Amresco, Solon, USA). The PCR reaction involved a 15 min Hot-Star Taq polymerase activation at 95°C followed by 45 cycles of denaturing at 94°C for 30 sec, annealing at 60°C for 30 sec and extension at 72°C for 30 sec, followed by a final extension for 10 min at 72°C. 

The specific G to T transversion at codon 249 of exon 7 was analyzed by Restriction Fragment Length Polymorphism (RFLP). PCR products were digested by *Hae*III restriction endonuclease (Promega, Madison, USA). The fragments were visualized on 3% agarose gel stained with ethidium bromide, eluted, reamplified, and sequenced by automated sequencing (sequencer 3730xl). Additionally, all samples were analyzed by direct sequencing of PCR products corresponding to *TP53* exons 7 and also exons 5, 6, and 8 as described elsewhere [26]. All results represent a minimum of two fully independent analyses. 

#### 2.2.6. Detection of HBV

HBx DNA sequences were amplified by PCR. Briefly, the PCR was carried out in a 20 *μ*L volume containing Colorless GoTaq flexi buffer, MgCl_2_ 1.5 mM, dNTP (200 *μ*M each), primers DG-XF4 (GGGACGTCCTTTGTCTACGT), and DG-X1R (GGGAGACCGCGTAAAGAGAG) and 0.8 U of GoTaq DNA polymerase (Promega). The PCR reaction involved a step at 95°C for 2 min followed by 50 cycles of denaturing at 94°C for 45 sec, annealing at 62°C for 45 sec and extension at 72°C for 45 sec, followed by a final extension for 7 min at 72°C. PCR products were analyzed by electrophoresis on 2% Agarose gels and ethidium bromide staining.

#### 2.2.7. HBV Genotyping

The small S gene fragment of HBV was amplified using YS1-YS2 in the first round, and s3-s3as (319 nt) in a second round [27, 28], or hep3–hep33 as a unique round of PCR [29]. All sequences obtained were compared with GenBank available sequences. Phylogenetic analyses by Neighbour Joining and Maximum Likelihood were conducted with MEGA 5.1; this program was also used for tree representation.

## 3. Results

### 3.1. Characteristic of Study Population

During the period 2000–2007, 192 HCC cases were diagnosed at Fundacion Santa Fe de Bogota (23.3%), Hospital Pablo Tobon Uribe (15.3%) and Facultad de Medicina, Universidad de Antioquia (56.4%). Additionally, 10 cases (5%) were diagnosed at Hospital Universitario del Valle during the period 2000–2004. 

Among the total HCC cases, 36% were diagnosed in females and 64% in males corresponding to a male/female ratio of 1.8 : 1. The average age was 62 years, the median was 61 years, and the age range was 22 to 90. The records of HCC by age showed a higher frequency starting in the sixth decade of life in both populations genders. No data from the cases were available on HCV or HBV status or alcohol consumption.

The 49 HCC cases included in this study were classified according to the Edmonson-Steiner criteria as G1 well-differentiated (4.9%), G2 moderately differentiated (39%), or G3 poorly differentiated (56.1%). The trabecular type was the most frequent (56.5%) followed by solid (21.7%), mixte (13%), glandular (4.4%), and pseudoglandular (4.4%). The clinicopathological characteristics of the HCC cases included in this study are summarized in Table 1.

### 3.2. Mutations in TP53 Gene

**Figure 1:**
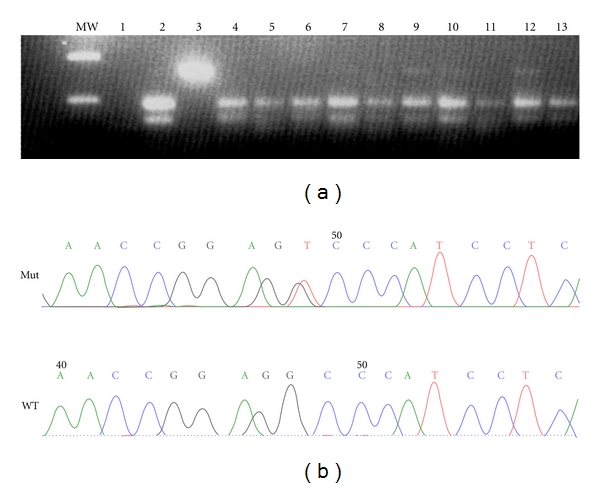
TP53 249^ser^ mutation in HCC cases from Colombia. (a) TP53 mutation at codon 249 was identified by restriction digestion. Presence of an undigested 158 bp fragment is indicative of mutation. Wild-type pattern: Lanes 2 (negative control: DNA from healthy donor lymphocytes), 4 to 8, 11, and 13 (HCC cases). Mutant pattern: Lanes 3 (positive control: DNA from PLC/PRF/5 cell line), 9, 10, and 12 (HCC cases). MW: molecular weight marker 100 bp. (b) Sequencing chromatograms of a mutant HCC case (a), showing the change from AGG to AGT and a wild-type HCC case (b).

The 249^ser^ mutation was investigated in HCC samples by RFLP followed by sequencing and by direct sequence of *TP53* exon 7 (Figure 1). The presence of a mutation was confirmed by both techniques in 4 (10.5%) of 38 HCC samples. The mutation was associated with overexpression of p53 in two of these samples (10%–50% of cells stained); for the other two cases, the immunochemistry analysis was not available. No additional mutations were detected in any other position of *TP53* exon 7. 

The analysis by direct sequence of TP53 exons 5, 6, and 8 revealed two point mutations in exon 5 (G12457T/V157F) and exon 8 (G13804A/C275Y) and one SNP in exon 6 (A12708G/R213R) in three (8%) HCC samples of the 34 without 249^ser^ mutation. No accumulation of p53 protein was demonstrated in the HCC sample exhibiting the mutation G12457T. The assay of p53 protein by immunochemistry was not available for the other two samples. 

### 3.3. AFB1-DNA Adducts

**Figure 2:**
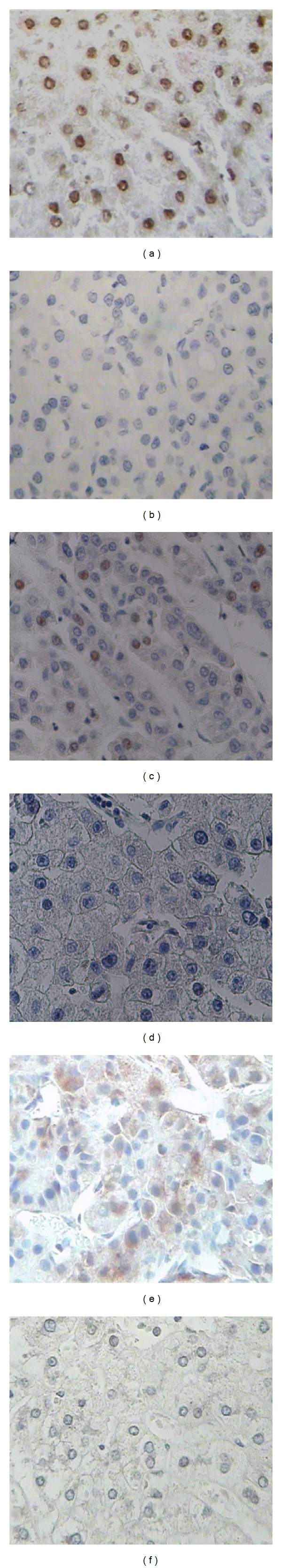
Immunohistochemical detection of HCC biomarkers. (a) Liver tissue specimens from Sprague-Dawley rats treated with 1.25 mg/Kg of AFB. (b) Liver sample from patient with HCC without apparent exposure to AFB. (c) HCC case with detection of HBx protein, high expression, and nuclear localization. (d) Negative HCC case to HBx protein detection. (e) HCC case with detection of HCV Core protein, high expression in the cytoplasm of liver cells. (f) HCC case without expression of HCV core protein. Original magnification ×400.

A total of 31 liver tissue samples were analyzed for AFB1-DNA adducts by immunohistochemistry. This biomarker was detected in hepatocyte nuclei of treated rat liver tissue included in each of the assays as a positive control; however, none of the HCC samples analyzed was positive for DNA adducts (Figures 2(a) and 2(b)). Twenty-three HCC samples were analyzed for both AFB1-DNA adducts and TP53 exon 7, but none of these samples was positive for the 249^ser^ mutation.

### 3.4. HBV Infection Biomarkers

Considering that data on the HBV infection status of the HCC cases analyzed was unavailable, two biomarkers were included in order to identify the cases associated with HBV infection. Twenty-five HCC samples (58.1%, 25/43) were positives for nuclear HBx protein by immunohistochemical detection (Figures 2(c) and 2(d)) and/or HBx sequence detection by PCR (nucleotides 1411–1549). From the 25 positives HCC cases, 5 samples were positive for both biomarkers; eleven samples were positives for one of HBV biomarkers (HBx immunohistochemical detection or HBx PCR), while 9 samples positive were analyzed for only one of the biomarkers.

The 249^ser^ and G13804A (exon 8) mutations were identified in two samples positive for HBV biomarkers. Regrettably, the analysis of HBV biomarkers was not available in one of the samples positive for the 249^ser^ mutation. 

### 3.5. HBV Genotypes

The small S gene fragment was analyzed by PCR and sequenced in 23 HCC samples positive for HBV biomarkers (HBx protein and/or HBx PCR). However, it was successfully sequenced in just three samples. One of the reasons for this limited number of samples characterized for the viral genotype could be the quality of the DNA extracted from the paraffin-fixed liver tissues. 

**Figure 3:**
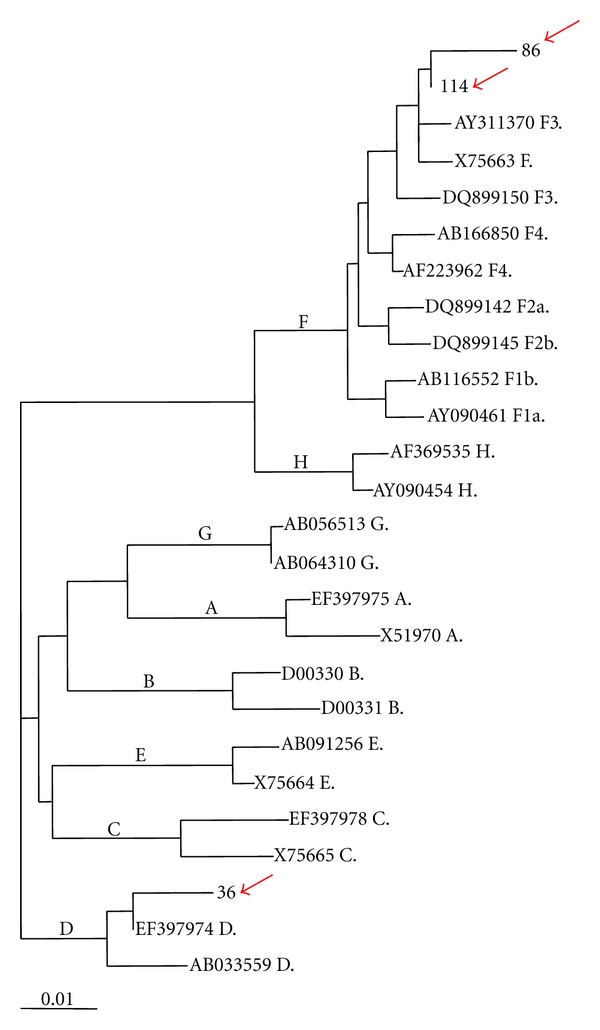
Phylogenetic tree of HBV genotypes generated by Neighbour joining method (MEGA), using HBV S gene sequences. The accesion number followed by genotype indentity is indicated. Bootstrap values are shown (1000 repetitions). HKY was used to access distances. Red arrows show the Colombian strain position.

Phylogenetic study of the S sequence showed that two isolates belonged to genotype F and one isolate to genotype D according to the grouping with HBV prototype GenBank sequences. Similar topology was observed between trees generated by the different inference methods (data not shown). The identification of the subtype was not available in these samples taking into account the limitations of the size sequence (Figure 3).

### 3.6. HCV Infection Biomarker

The Core HCV protein was detected by immunohistochemistry in 37% of the samples analyzed (10/27); cytoplasmic staining was observed in all positive HCC samples (Figures 2(e) and 2(f)). HBV/HCV coinfection was demonstrated in 19.2% (5/26) of the liver tissue samples included in the assays; two were positive for both biomarkers of HBV and HCV infection, while the other three were positive for HBx by immunohistochemical detection or HBx PCR, in addition to the Core HCV protein detection. 

## 4. Discussion

This is the first study of HCC biomarkers carried out in a Colombian population. The health centers of this study included two leading Departments of Pathology at the national level (Fundacion Santa Fe de Bogota and Facultad de Medicina, Universidad de Antioquia) and two of the most important hospitals in Medellin and Cali Cities (Hospital Pablo Tobon Uribe and Hospital Universitario del Valle). 

Although, the 202 cases recruited in these centers during the period 2000–2007 do not represent the national registries, the data obtained from these reference institutions contributes to the knowledge of HCC epidemiology of Colombia.

The burden of HCC has demonstrated an increasing trend over the past two decades in some regions of the world [1]. Even in Latin America, previously considered as a low incidence of liver cancer region, the data from different countries revealed increasing rates of HCC. Indeed, the mortality rates for primary liver cancer have increased in Mexico from 4.1/100.000 inhabitants in 2000 to 4.7/100.000 in 2006 [30]. A similar tendency is observed in Colombia; the mortality rates for primary liver and intrahepatic bile duct cancer were 4.8/100.000 inhabitants in 2000 and 5.0/100.000 inhabitants in 2001 [5, 31]. Moreover, the mortality rates for these cancers in Antioquia state increased from 6.9/100.000 in 2003 to 33/100.000 in 2005 [32]. The increased trend in mortality rates for the country and for Antioquia state could be related to improved diagnostic procedures in the health system. On the other hand, changes in risk factors over time could also be implicated. 

The analysis of GLOBOCAN data revealed an overall male : female ratio of 2.4 : 1. The reported ratios usually varied between 2 : 1 to 4 : 1 depending on the incidence rates and risk factor patterns over the world. The higher rates of liver cancer in male population could be due to differences in risk factor exposure [33]. 

Central and South America have the lowest reported male : female ratio for liver cancer; for example, 1.2 : 1 in Colombia and 1.6 : 1 in Costa Rica [33]. However, as mentioned previously the data sources from Colombia in the GLOBOCAN database are restricted to the cancer registry of Cali city. In this study, from the 202 HCC cases, diagnosed at 4 institutions in Bogota, Medellin, and Cali cities, the male : female incidence ratio was 1.8 : 1.

In low-risk populations, the highest age-specific rates arise in patients around 75 years old while in high-risk population it occurs around 60 to 65 years old. The age-specific pattern is related to differences in HBV and HCV prevalence, age of infection, and other relevant risk factors in a population [33]. The mean age of the 202 HCC cases of this study was 62 years and the median 61 years. These data are similar to the median age in other studies carried out in Peru [34] and Argentina [13] and also in a multicenter prospective HCC study in 9 Latin American countries [6]. Nevertheless, in other studies the mean age of HCC patients was 41.4 years in the Peruvian population [35], 55.9 years in the Brazilian population [12], and 56 years in the Chilean population [14]. The mean age difference among the HCC studies in Latin American countries could be related to the risk factor patterns in each country.

According to the IARC TP53 database [36] (R15, http://www-p53.iarc.fr), TP53 mutations have been described in up to 31.4% of HCC cases, with the 249^ser^ mutation being the most common. This mutation has been associated with AFB1 exposition and there is robust evidence that supports this finding. AFB1 is classified as an IARC Group 1 carcinogen for the liver and causes an inactivating mutation at codon 249 of the *TP53* tumor suppressor gene, inducing the substitution of an arginine by a serine (*R249S *mutation) [1].

This mutation has a high frequency in populations from the highest-HCC-incidence areas like Qidong, China (43.8%) [37], Guangxi, China (36%) [38], and Gambia (39.8%) [39]. Moreover, 71.5% of reported mutations in codon 249 of TP53, which correspond to the transversion G : C → T : A have been detected in HCC [36], whereby this mutation could be a biomarker for dietary exposure to anatoxin.

In this study, the 249^ser^ mutation was detected in 10.5% (4/38) of the HCC samples. A similar prevalence was reported in other areas in Anhui, Province of China (10.5%) [40], India (9.5%) [41], and Taiwan (13%) [42]; in recent studies a prevalence around 2% was reported in Turkey [43] and in Taiwan [44]. The main risk factor of HCC in these studies was HBV infection, similar to that reported in this Colombian population. 

The 249^ser^ mutation frequency described for the first time in a Colombian population suggests an AFB1 exposure level between low to intermediate. Three of the four HCC samples positive for this mutation were cases recruited in one of the most important reference national health center (Fundacion Santa fe de Bogota); probably these samples corresponded to HCC cases from rural areas. 

A correlation between AFB1 adducts and the 249^ser^ was demonstrated in two studies carried out in Taiwan [42, 44]. However, none of the HCC cases included in our study were positive for AFB1-DNA adducts, even though there is evidence of AB1 contamination in corn and rice collected in supermarkets, retail stores, and stock centers in Colombia [19]. 

Until now, there are only two studies published regarding the 249^ser^ mutations in HCC cases in Latin American countries. One of them was carried out in Mexico in 16 HCC samples with a 249^ser^ frequency of 19% [45]. The other one was carried out in Brazil, where the maximum AFB1 level in food allowed is higher than in North America and Europe; the 249^ser^ prevalence was 28% (21/74) by PCR-RFLP and 16% by direct sequencing [46]. The 249^ser^ prevalence data of 16% in Brazil and 10.5% in Colombia using both techniques (RFLP and direct sequence) for mutation analysis suggest that dietary exposure to AFB1 is an HCC risk factor in these Latin America countries, although not as important as in Africa and Asia. 

Different studies have suggested that 249^ser^ mutation occurs almost exclusively in the context of chronic HBV infection in addition to AFB1 chronic dietary exposure [1]. Kirk et al. demonstrated in a prospective study in Gambia, a multiplicative effect on HCC risk resulting from HBV chronic infection and the mutational effect of AFB1 on the TP53 gene (codon 249). Actually, in 216 HCC incident cases and 121 cirrhosis cases, the risk for HCC was associated with HBV markers with an odds ratio (OR) of 10.0 (95%, CI: 5.16–19.6), 249^ser^ with an OR of 13.2 (95%, CI: 4.99–35.0), and both markers with an OR of 300 (95%, CI: 48.6–3270) [39]. In this study of a Colombian population, the analysis of HBV biomarkers and TP53 exon 7 sequences was performed in 32 HCC samples, including 3 from 4 249^ser^ mutation-positive samples. One of three samples was positive for both biomarkers (HBV and 249^ser^). The negative results for HBV biomarkers in the other two samples could be explained by technical limitations of immunohistochemistry and PCR protocols used in this study. However, Kirk et al. have described 15.3% of HCC samples HBV(−)/TP53 249^ser^(+) [39]. Additionally, we report two point mutations, G12457T (exon 5) and G13804A (exon 8), located in the p53 DNA-binding domain. These missense mutations modify the stability and transactivating properties of p53 protein; according to the TP53 database, their frequency in HCC is 2.1 and 0.26%, respectively [36]. In this study, the mutations were described in two HCC cases (2.9%, 1/34, each one).

HBV biomarkers were demonstrated in 58.1% of the HCC samples analyzed in this study by HBx protein immunohistochemistry detection and HBx PCR. These data are expected considering the epidemiological pattern of HBV infection in Colombia, average moderate prevalence but with regions of high prevalence [47, 48]. A higher frequency of HBV infection than other risk factors has also been described in HCC patients from Peru (38.9%, 42.2%) [34, 35] and in states of the north eastern and northern regions of Brazil (average 43.1%), including Para (71.4%, 5/7), Bahia (45%, 9/20), Minas Gerais (37.8%, 14/37), and Espirito Santo (41.6%, 10/24) [12]. HBV infection was diagnosed in these studies by the detection of HBsAg in serum samples from the patients. As mentioned previously, data on HBsAg in the Colombian patients were not available from the clinical records.

The phylogenetic study of the S sequence in three HCC samples in this study was successful. Two isolates were characterized as genotype F and one as genotype D. The HBV genotypes in the Colombian population have been characterized in four studies in blood donor populations, pregnant women, and recently in end-stage liver diseases cases. The predominance of genotype F was demonstrated in three of these studies (77%, 87.23%, and 100%) [49–51]. Additionally, genotypes A, D, C, G, and E were also described in some cases of those studies [49, 50, 52]. 

The HCV infection status was established by HCV Core protein immunohistochemistry detection. Regrettably, molecular biology markers for HCV infection were not available. A prevalence of 37% was found in the HCC samples analyzed in this study with an HBV/HCV coinfection prevalence of 19.2%. In other Latin American countries where the HBV infection is the most important HCC risk factor, the prevalence of the HCV marker is lower (5.3%–16.6%) than described in this Colombian population. Nevertheless, the HCV biomarker in these studies carried out in Peru [34, 35] and Brazil [12] corresponded to anti-HCV detection; unfortunately, data on this biomarker were not available for the Colombian population in this study. 

 On the other hand, HCV infection is the predominant HCC risk factor in Argentina, Chile, and the South eastern states of Brazil (Rio de Janeiro, Sao Paulo, Parana, and Rio Grande do Sul). The prevalence range of the serum anti-HCV marker was 32.8% to 44% in HCC cases from these South American countries [12–14]. Furthermore, in a recent prospective multicenter study of HCC cases from 9 Latin American countries, including Argentina, Brazil, Chile, Colombia, Uruguay, and Venezuela, the main HCC risk factor was HCV infection (30.8%), followed by alcohol (20.4%), HBV infection (10.8%), and HCV plus alcohol (5.8%) [6]. Some cases from this multicenter study corresponded to patients recruited in a prospective study carried out at Hospital Pablo Tobon Uribe (HPTU) in Medellin city; Interestingly, from the total 131 cases of end-stage liver disease in this hospital in the period of the study, the most important risk factor was chronic alcoholism (37.4%, 49/131), whereas viral infection ranked second (10.7% HBV and 6.9% HCV); the serum markers for diagnosis of viral infections were HBsAg and anti-HCV [51]. Chronic alcoholism was also an important risk factor in HCC patients in Argentina (42%), Brazil (37%), and Chile (31%) [12–14]. These results suggest that the HCC risk factors pattern in Latin America is changing to the pattern seen in developed countries, where one of the principle HCC risk factors is chronic alcohol abuse [1]. However, further investigations are necessary to confirm this hypothesis.

Unfortunately, only demographic and histopathological data but not alcohol intake were available from the HCC cases in this study. The discrepancy between the risk factor pattern revealed in this retrospective study carried out in four Departments of Pathology from health institutions in Cali, Medellin, and Bogota cities and in the prospective one performed at hepatology unit of HPTU in Medellin city could be partially explained by differences in age range, male : female ratio, viral infection biomarkers, and quality of clinical information, including follow-up of the patients in the second study. However, the predominance of HBV infection compared to HCV infection was described in both populations. Further case-control studies in the Colombian population are necessary in order to define the burden of alcohol intake and viral infections as HCC risk factors.

In conclusion, this first retrospective study exploring the epidemiological features of HCC in patients from 4 leading health institutions in the three most important cities in Colombia revealed that the majority of the patients are male and in their 6th to 7th decade of life. The main HCC risk factor is HBV infection but the presence of codon 249 mutations, a biomarker of AFB1 exposure, was also found in some HCC samples. 

## Figures and Tables

**Table 1:** Clinicopathological data of HCC cases diagnosed at four health reference institutions during the period 2000–2007 in Colombia.

	Frequency** (%) ** *n* = 49
Mean age	63.5 years
Age range	25–88 years
Gender female/male	43.2%/56.8%
Edmondson and Steiner's grade	
G1	4.9%
G2	39%
G3	56.1%
Histological type	
Trabecular	56.5%
Solid	21.7%
Mixte	13%
Glandular	4.4%
Pseudo-glandular	4.4%
*Biomarkers*	
HBV	58.1% (25/43)
HCV	37% (10/27)
Coinfection	19.2% (5/26)
AFB1-DNA adducts	0% (0/31)
*TP53 gene *	
Exon 7 (249^ser^ mutation)	10.5% (4/38)
Exon 5 (G12457T mutation)	2.9% (1/34)
Exon 8 (G13804A mutation)	2.9% (1/34)

## References

[1] Peter P, Levin B (2008). *World Cancer Report 2008*.

[2] Perz JF, Armstrong GL, Farrington LA, Hutin YJ, Bell BP (2006). The contributions of hepatitis B virus and hepatitis C virus infections to cirrhosis and primary liver cancer worldwide. *Journal of Hepatology*.

[3] Venook AP, Papandreou C, Furuse J, de Guevara LL (2010). The incidence and epidemiology of hepatocellular carcinoma: a global and regional perspective. *The Oncologist*.

[4] International Agency for Research on Cancer (2007). *Cancer Incidence in Five Continents*.

[6] Fassio E, Díaz S, Santa C (2010). Etiology of hepatocellular carcinoma in Latin America: a prospective, multicenter, international study. *Annals of Hepatology*.

[7] Añez MS, Zabaleta P, Umbria L (1986). Carcinoma hepatocellular en los Andes Venezolanos. *GEN*.

[8] Mondragón R, Ochoa F, Ruiz J, Goepfer RH, Ocana LFO, Crocifoglio VA (1997). Carcinoma hepatocelular. Experiencia en el Instituto Nacional de Cancerología. *Revista de Gastroenterologia*.

[9] Curciarello JO, Vélez LD, Bosia JD (1998). Carcinoma hepatocelular: características clinicoepidemiológicas. *Acta Gastroenterologica Latinoamericana*.

[10] Bueno HR, Indacochea S, Oberst RB, Chauca G (1994). Rol de los virus de la hepatitis B and Delta en la etiología del hepatocarcinoma y otras crónicas. *Revista de Gastroenterologia del Peru*.

[11] Mattos AA, Machado SB, Lucas ML, Zettler CG (1999). Expresao da proteína mutente do gene supressor tumoral p53 em carcinoma hepatocelular no Sul do brasil: estudo imuno-histoquimico. *Gastroenterologia Endoscopia Digestive*.

[12] Gonçalves CS, Pereira FE, Gayotto LC (1997). Hepatocellular carcinoma in Brazil: report of a national survey (Florianópolis, SC, 1995). *Revista do Instituto de Medicina Tropical de Sao Paulo*.

[13] Fassio E, Míguez C, Soria S (2009). Etiology of hepatocellular carcinoma in Argentina: results of a multicenter retrospective study. *Acta Gastroenterologica Latinoamericana*.

[14] Gabrielli M, Vivanco M, Hepp J (2010). Liver transplantation results for hepatocellular carcinoma in Chile. *Transplantation Proceedings*.

[15] de la Hoz F, Perez L, Wheeler JG, de Neira M, Hall AJ (2005). Vaccine coverage with hepatitis B and other vaccines in the Colombian Amazon: do health worker knowledge and perception influence coverage?. *Tropical Medicine and International Health*.

[16] de la Hoz F, Perez L, de Neira M, Hall AJ (2008). Eight years of hepatitis B vaccination in Colombia with a recombinant vaccine: factors influencing hepatitis B virus infection and effectiveness. *International Journal of Infectious Diseases*.

[17] Beltrân M, Navas MC, de la Hoz F (2005). Hepatitis C virus seroprevalence in multi-transfused patients in Colombia. *Journal of Clinical Virology*.

[18] Duarte-Vogel S, Villamil-Jiménez LC (2006). Micotoxinas en salud pública. *Revista de Salud Pública*.

[19] Diaz GJ, Perilla NS, Rojas Y (2001). Ocurrence of aflatoxins in selected Colombian foods. *Mycotoxin Research*.

[20] Gaviria I, Giraldo J (1995). *Evaluación Genotóxica de Aflatoxinas Aisladas de Maíz Almacenado para Consumo Humano y Animal*.

[21] Carpintero M, Ruiz N, Peña N (1980). Niveles de aflatoxina B1 en dos zonas del país. Primera cosecha de 1979. *Revista ICA*.

[22] Cárdenas O (1986). Contaminación de aflatoxinas en productos agrícolas nacionales. Sorgo del Meta. *Istituto Italiano di Tecnologia*.

[23] Prado G (1983). Incidencia de aflatoxina B1 em alimentos. *Revista de Farmácia e Bioquímica*.

[24] Hsieh LL, Hsu SW, Chen DS, Santella RM (1988). Immunological detection of aflatoxin B1-DNA adducts formed *in vivo*. *Cancer Research*.

[25] Szymánska K, Lesi OA, Kirk GD (2004). Ser-249 TP53 mutation in tumour and plasma DNA of hepatocellular carcinoma patients from a high incidence area in the Gambia, West Africa. *International Journal of Cancer*.

[26] Hosny G, Farahat N, Hainaut P (2009). TP53 mutations in circulating free DNA from Egyptian patients with non-Hodgkin’s lymphoma. *Cancer Letters*.

[27] Zeng GB, Wen SJ, Wang ZH, Yan L, Sun J, Hou JL (2004). A novel hepatitis B virus genotyping system by using restriction fragment length polymorphism patterns of S gene amplicons. *World Journal of Gastroenterology*.

[28] Schaefer S, Glebe D, Wend UC, Oyunbileg J, Gerlich WH (2003). Universal primers for real-time amplification of DNA from all known orthohepadnavirus species. *Journal of Clinical Virology*.

[29] Norder H, Hammas B, Lee SD (1993). Genetic relatedness of hepatitis B viral strains of diverse geographical origin and natural variations in the primary structure of the surface antigen. *Journal of General Virology*.

[30] Méndez-Sánchez N, Villa AR, Vázquez-Elizondo G, Ponciano-Rodríguez G, Uribe M (2008). Mortality trends for liver cancer in Mexico from 2000 to 2006. *Annals of Hepatology*.

[31] Jaramillo FLO, Vélez LPM (2004). Mortalidad por cáncer en Colombia 2001. *CES Medicina*.

[32] Dirección Seccional de Salud de Antioquia Registro poblacional de cáncer. Diez primeras causas de mortalidad por grupos de edad según 105 grupos. http://www.dssa.gov.co/index.php/estadisticas/registro-poblacional-de-cancer.

[33] El-Serag HB, Rudolph KL (2007). Hepatocellular carcinoma: epidemiology and molecular carcinogenesis. *Gastroenterology*.

[34] Sánchez CB, Ferrer JD, Vargas RR, Moscol MD, Villena EZ (2009). Clinical—epidemiological characteristics of the hepatocellular carcinoma and treatment in the departament of digestive system diseases of the National Hospital “Eduardo Rebagliatti Martins” (HNERM)—ESSALUD. *Revista de Gastroenterología del Perú*.

[35] Ruiz E, Sanchez J, Celis J (2007). Short and long-term results of liver resection for hepatocarcinoma in Peru: a Peruvian single center experience on 232 cases. *Revista de Gastroenterologia del Perú*.

[36] Petitjean A, Mathe E, Kato S (2007). Impact of mutant p53 functional properties on TP53 mutation patterns and tumor phenotype: lessons from recent developments in the IARC TP53 database. *Human Mutation*.

[37] Hsu IC, Metcalf RA, Sun T, Welsh JA, Wang NJ, Harris CC (1991). Mutational hotspot in the p53 gene in human hepatocellular carcinomas. *Nature*.

[38] Stern MC, Umbach DM, Yu MC, London SJ, Zhang ZQ, Taylor JA (2001). Hepatitis B, aflatoxin B1, and p53 codon 249 mutation in hepatocellular carcinomas from Guangxi, People’s Republic of China, and a Meta-analysis of existing studies. *Cancer Epidemiology Biomarkers and Prevention*.

[39] Kirk GD, Lesi OA, Mendy M (2005). 249ser TP53 mutation in plasma DNA, hepatitis B viral infection, and risk of hepatocellular carcinoma. *Oncogene*.

[40] Liu H, Wang Y, Zhou Q, Gui SY, Li X (2002). The point mutation of p53 gene exon7 in hepatocellular carcinoma from Anhui province, a non HCC prevalent area in China. *World Journal of Gastroenterology*.

[41] Katiyar S, Dash BC, Thakur V, Guptan RC, Sarin SK, Das BC (2000). p53 tumor suppressor gene mutations in hepatocellular carcinoma patients in India. *Cancer*.

[42] Lunn RM, Zhang YJ, Wang LY (1997). p53 mutations, chronic hepatitis B virus infection, and aflatoxin exposure in hepatocellular carcinoma in Taiwan. *Cancer Research*.

[43] Özdemir FT, Tiftikci A, Sancak S (2010). The prevalence of the mutation in codon 249 of the P53 gene in patients with hepatocellular carcinoma (HCC) in Turkey. *Journal of Gastrointestinal Cancer*.

[44] Zhang YJ, Rossner P, Chen Y (2006). Aflatoxin B1 and polycyclic aromatic hydrocarbon adducts, p53 mutations and p16 methylation in liver tissue and plasma of hepatocellular carcinoma patients. *International Journal of Cancer*.

[45] Soini Y, Chia SC, Bennett WP (1996). An aflatoxin-associated mutational hotspot at codon 249 in the p53 tumor suppressor gene occurs in hepatocellular carcinomas from Mexico. *Carcinogenesis*.

[46] Nogueira JA, Ono-Nita SK, Nita ME (2009). 249 TP53 mutation has high prevalence and is correlated with larger and poorly differentiated HCC in Brazilian patients. *BMC Cancer*.

[47] Sivigila (2006). Sistema de vigilancia en salud pública. *Inf Quinc Epidemiol Nac*.

[48] Sivigila (2006). Sistema de vigilancia en salud pública. *Inf Quinc Epidemiol Nac*.

[49] Devesa M, Loureiro CL, Rivas Y (2008). Subgenotype diversity of hepatitis B Virus American genotype F in amerindians from Venezuela and the general population of Colombia. *Journal of Medical Virology*.

[50] Mora MVA, Romano CM, Gomes-Gouvêa MS (2011). Molecular characterization of the hepatitis B virus genotypes in Colombia: a bayesian inference on the genotype F. *Infection, Genetics and Evolution*.

[51] Cortes-Mancera F, Loureiro CL, Hoyos S (2011). Etiology and viral genotype in patients with end-stage liver diseases admitted to a Hepatology unit in Colombia. *Hepatitis Research and Treatment*.

[52] Mora MVA, Romano CM, Gomes-Gouvêa MS, Gutierrez MF, Carrilho FJ, Pinho JRR (2010). Molecular epidemiology and genetic diversity of hepatitis B virus genotype E in an isolated Afro-Colombian community. *Journal of General Virology*.

